# Outcomes of dual-mobility total hip arthroplasty versus bipolar hemiarthroplasty for patients with femoral neck fractures: a systematic review and meta-analysis

**DOI:** 10.1186/s13018-021-02316-6

**Published:** 2021-02-24

**Authors:** Hsuan-Hsiao Ma, Te-Feng Arthur Chou, Fu-Yuan Pai, Shang-Wen Tsai, Cheng-Fong Chen, Po-Kuei Wu, Wei-Ming Chen

**Affiliations:** 1 Department of Orthopaedics and Traumatology, Taipei Veterans General Hospital, No. 201, Sec 2, Shi-Pai Road, Taipei, 112 Taiwan; 2 Department of Orthopaedics, School of Medicine, National Yang-Ming University, Taipei, Taiwan

**Keywords:** Dislocation, Dual mobility, Elderly, Femoral neck fracture, Hemiarthroplasty, Total hip arthroplasty, Hip fracture, Reoperation

## Abstract

**Background:**

Elderly patients with femoral neck fractures are at a higher risk of dislocation after hip arthroplasty procedures. In comparison with total hip arthroplasty (THA), bipolar hemiarthroplasty (HA) and dual-mobility total hip arthroplasty (DM-THA) can be an effective alternative treatment which increases the effective head size and overall stability of the prosthesis. We aim to review the current evidence on the outcome after DM-THA and HA for femoral neck fractures in the elderly.

**Methods:**

We performed a comprehensive review of literatures on PubMed, Embase, Web of Science, and the Cochrane Library for randomized controlled trials and comparative interventional studies. Of the 936 studies identified, 8 met the inclusion criteria (541 DM-THA and 603 HA procedures). Two reviewers independently reviewed and graded each study and recorded relevant data including dislocation rate, implant failure rate, reoperation rate, 1-year mortality rate, Harris hip score (HHS), operation time, and intraoperative blood loss.

**Results:**

DM-THA was associated with a lower dislocation rate (OR 3.599; 95% CI 1.954 to 6.630), a lower reoperation rate (OR 2.056; 95% CI 1.211 to 3.490), an increased operation time (SMD − 0.561; 95% CI − 0.795 to − 0.326) and more intraoperative blood loss (SMD − 0.778; 95% CI − 1.238 to − 0.319), compared with the HA group. Moreover, the multivariate regression analysis revealed that age, female sex, posterolateral surgical approach, and choice of DM-THA or HA were not associated with dislocation or reoperation.

**Conclusions:**

Based on the current evidence, the advantages reported for DM-THA over HA with regard to dislocation and reoperation rate in elderly patients with FNF remain inconclusive. High-quality studies on the high-risk patients with cognitive disorder or dementia are necessary to validate the value of DM-THA.

**Supplementary Information:**

The online version contains supplementary material available at 10.1186/s13018-021-02316-6.

## Background

Hemiarthroplasty (HA) and total hip arthroplasty (THA) are treatment options for elderly patients with femoral neck fractures (FNF) [1]. Compared with THA, HA is associated with a lower dislocation rate but many studies have reported lower functional performance and higher reoperation rates [2, 3]. In recent years, dual-mobility THA (DM-THA) has been shown to be an effective treatment for FNF. In comparison with standard THA, the design of DM-THA increases effective head size which can decrease the rate of dislocation in both primary and revision THA [4]. The development of DM-THA has been a promising prosthesis for the elderly population with FNF since these patients often have increased dislocation rates after standard THA [2, 3, 5]. Moreover, elderly patients frequently have a frail status [6–8]. Therefore, reducing reoperation rates are essential in this population.

Currently, the outcome of HA and DM-THA procedures in elderly patients with FNF have been inconclusive [9–15]. Several studies have reported a lower dislocation rate in the DM-THA group [11, 14, 15], while others did not find a difference [9, 13]. In terms of patient-reported outcome, some studies have concluded that DM-THA was a more favorable operation with higher postoperative Harris hip scores (HHS) after surgery [10, 12, 13]. However, Nonne et al. reported similar scores in both groups [9]. Due to these inconclusive results, we conducted a meta-analysis to determine the outcome for elderly patients that underwent either HA or DM-THA for FNF. We hypothesize that patients who received DM-THA are associated with a lower dislocation and reoperation rates and improved postoperative functional score compared with patients that underwent HA.

## Methods

### Search strategy

**Fig. 1:**
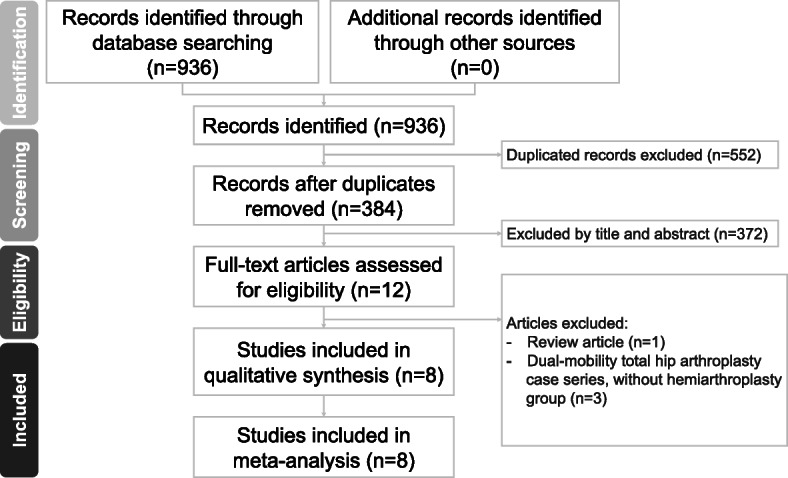
Preferred Reporting Items for Systematic reviews and Meta-analysis (PRISMA) flow diagram for the searching and identification of included studies.

We performed a literature search on PubMed, Embase, Web of Science, and the Cochrane library to identify relevant studies from the earliest record to April, 2020. The bibliographies of the included studies were manually reviewed for relevant references. We recorded studies that compared the outcomes of DM-THA and HA procedures for patients with FNF. The search strategy comprised the following keywords in variable combination: (femoral neck fracture OR femur neck fracture) AND (dual mobility OR hemiarthroplasty OR hip arthroplasty). Of the types of included studies, we enrolled randomized controlled trials (RCTs), prospective or retrospective comparative interventional studies. We excluded single-armed case series or case reports, studies that were not available in full text or not written in English. All identified studies comprised two treatment arms, one of which was DM-THA and the other was HA. The search strategy is presented in Fig. 1.

### Inclusion criteria

Studies were considered eligible if they met the PICOS (population, intervention, comparator outcomes, study design) criteria. Population includes patients with FNF. Intervention includes DM-THA or HA procedure as the surgical method for FNF. Comparator is DM-THA or HA procedure. Outcomes are dislocation rate, implant failure rate, reoperation rate, 1-year mortality rate, HHS, operation time, and intraoperative blood loss. Studies must have a follow-up rate of at least 90%, and at least one of the above outcome domains must be included. We included RCTs and comparative interventional studies.

### Data extraction and quality assessment

Two reviewers (SW-T, HH-M) examined all the identified studies and extracted data using a predetermined form. We recorded the first author, year, study design, enrolled sample number, age, sex, surgical treatment methods, mean follow-up duration, dislocation rate, implant failure rate, reoperation rate, 1-year mortality rate, HHS, operation time, and intraoperative blood loss (Table 1). Two reviewers independently evaluated the methodological quality of the enrolled studies using the modified Jadad Scale [17] to reduce bias and to ensure our results were reliable and veritable (Table 2). Discrepancies between the two reviewers were solved after thorough discussion. Funnel plots were constructed to visually detect the presence of publication bias (Fig S1, S2, S3, S4, S5, S6 and S7).

**Table 1:** Characteristics of included studies

Author, year	Study design	Enrolled sample number (G1/G2)	Age (G1/G2)	Female sex (G1/G2)	Comparing	Mean follow-up	Surgical approach	Single or multi-surgeon	Outcome measurement						
									a	b	c	d	e	f	g
Nonne, 2019 [9]	Retrospective comparative study	88/60	86.1/87.6	66(75%)/45(75%)	HA vs. DM	28.3 months	PL	Not mentioned	V	V			V		
Ukaj, 2019 [10]	Randomized controlled trial	32/34	77.6/78.1	15(32%)/24(51%)	HA vs. DM	36 months	PL	Single surgeon	V	V		V	V	V	V
Iorio, 2019 [11]	Randomized controlled trial	30/30	83/82	17(57%)/18(60%)	HA vs. DM	12 months	DL	Not mentioned	V	V	V	V		V	
Fahad, 2019 [12]	Retrospective comparative study	77/27	71.1/69.3	46(60%)/14(52%)	HA vs. DM	12 months	DL or PL	Multi-surgeon		V		V	V		
Kim, 2018 [13]	Retrospective comparative study	84/84	72.9/73.1	57(68%)/58(69%)	HA vs. DM	21.9 months	PL	Single surgeon	V			V	V	V	V
Boukebous, 2018 [14]	Retrospective comparative study	101/98	83.3/77.8	73(73%)/70(71%)	HA vs. DM	24.6 months	PL	Multi-surgeon	V		V			V	
Ochi, 2017 [16]	Retrospective comparative study	20/33	75.4/80.0	16(75%)/26(78%)	HA vs. DM	20.5 months	DAA	Multi-surgeon	V	V	V	V		V	V
Bensen, 2014 [15]	Retrospective comparative study	171/175	84.1/75.2	131(77%)/123(70%)	HA vs. DM	23.5 months	PL	Multi-surgeon	V	V	V	V		V	V

G1: *HA* hemiarthroplasty; G2: *DM* dual-mobility total hip arthroplasty; *DAA* direct anterior approach; *DL* direct lateral approach; *PL* posterolateral approach; *a* dislocation; *b* Implant failure; *c* reoperation; *d* 1-year mortality; *e* Harris hip score; *f* operation time; *g* intraoperative blood loss

**Table 2:** Quality assessment of the included studies by modified Jadad Scale

Item assessed	Nonne, 2019 [9]	Ukaj, 2019 [10]	Iorio, 2019 [11]	Fahad, 2019 [12]	Kim, 2018 [13]	Boukebous, 2018 [14]	Ochi, 2017 [16]	Bensen, 2014 [15]
Was the study described as randomized?	No	Yes	Yes	No	No	No	No	No
Was the method of randomization appropriate?	No	Yes	Yes	No	No	No	No	No
Was the study described as blinded?	No	Yes	Yes	No	No	No	No	No
Was the method of blinding appropriate?	No	Yes	Yes	No	No	No	No	No
Was there a description of withdrawals and dropouts?	Yes	Yes	Yes	Yes	Yes	Yes	Yes	Yes
Was there a clear description of the inclusion/exclusion criteria?	Yes	Yes	Yes	Yes	Yes	Yes	Yes	Yes
Was the method used to assess adverse effects?	Yes	Yes	Yes	Yes	Yes	Yes	Yes	Yes
Was the method of statistical analysis described?	Yes	Yes	Yes	Yes	Yes	Yes	Yes	Yes
Scores	4	8	8	4	4	4	4	4

### Data synthesis

The odds ratio (OR) of the dislocation rate, implant failure rate, and reoperation rate between the DM-THA and HA group were the primary outcomes. The OR of 1-year mortality, the standardized mean differences (SMDs) of HHS at the last follow-up, operation time, and intraoperative blood loss were the secondary outcomes. An OR value less than 1 or a negative SMD value indicated that HA procedure was a favorable treatment option. A random effect model was utilized to pool individual SMDs and ORs. A standard multivariable linear regression analysis (*β*) was performed for potential risk factors for dislocation and reoperation rate. Analyses were performed using Comprehensive Meta-Analysis (CMA) software, version 3 (Biostat, Englewood, NJ, USA). Between-trial heterogeneity was determined by using *I*^2^ tests; values > 50% were regarded as considerable heterogeneity. Statistical significance was defined as *p* values < 0.05.

## Results

### Search results

According to our search strategy, we identified 936 relevant studies. We removed 552 duplicate records using the Endnote software. We excluded 372 studies after reading the title and abstract. Four studies that did not meet the inclusion criteria were excluded after reading the full text. Finally, 8 studies that compared DM-THA and HA for patients with FNF were included for analysis [9–16] (Fig. 1). The baseline characteristics of the 8 studies are summarized in Table 1. A total of 2 RCTs and 6 retrospective comparative studies were included.

### Methodological quality assessment

All of the included studies were good to excellent quality based on the modified Jadad Scale (Table 2). Two articles scored 8 and the other six articles scored 4. These six articles were graded lower since the study design was a retrospective comparative study. However, the six studies had clear descriptions of the withdrawals and dropouts, inclusion and exclusion criteria, adverse effects, and statistical analysis.

### Meta-analysis results

#### Dislocation rate

**Fig. 2:**
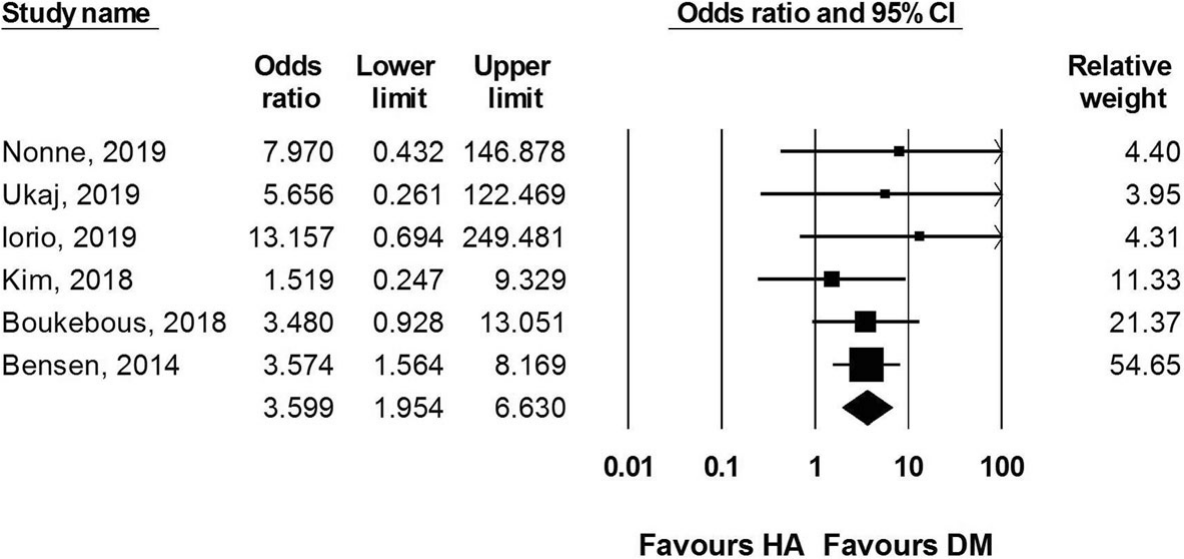
Forest plot comparing dislocation rate after dual-mobility total hip arthroplasty (DM-THA) versus bipolar hemiarthroplasty (HA)

Seven studies reported the dislocation rates after DM-THA and HA surgery. A total of 541 DM-THA and 603 HA procedures were included. Ochi et al. did not report any dislocation event in either DM-THA or HA group [16]. Our results revealed a higher dislocation rate after HA than after DM-THA with an OR of 3.599 (95% CI 1.954 to 6.630, *I*^2^ = 0, Fig. 2). The multivariate regression analysis revealed that age, female sex, posterolateral surgical approach, and choice of DM-THA or HA were not associated with dislocation (Table 3).

**Table 3:** Multivariate linear regression analysis

Independent variable	*β*-coefficient	95% confidence interval	*P* value
Dislocation			
Age	0.06	− 0.05–0.18	0.228
Female Sex	− 1.35	− 9.76–7.07	0.753
Posterolateral approach (ref to others)	0.18	− 0.59–0.94	0.857
Surgery (ref to HA)			
DM-THA	− 0.89	− 2.02–0.24	0.125
Reoperation			
Age	− 0.16	− 0.32–0.01	0.070
Female Sex	− 3.01	− 10.89–4.86	0.453
Posterolateral approach (ref to others)	1.09	− 0.49–2.67	0.176
Surgery (ref to HA)			
DM-THA	− 1.37	− 2.83–0.1	0.067

*DM-THA* dual-mobility total hip arthroplasty; *HA* bipolar hemiarthroplasty

#### Implant failure rate

**Fig. 3:**
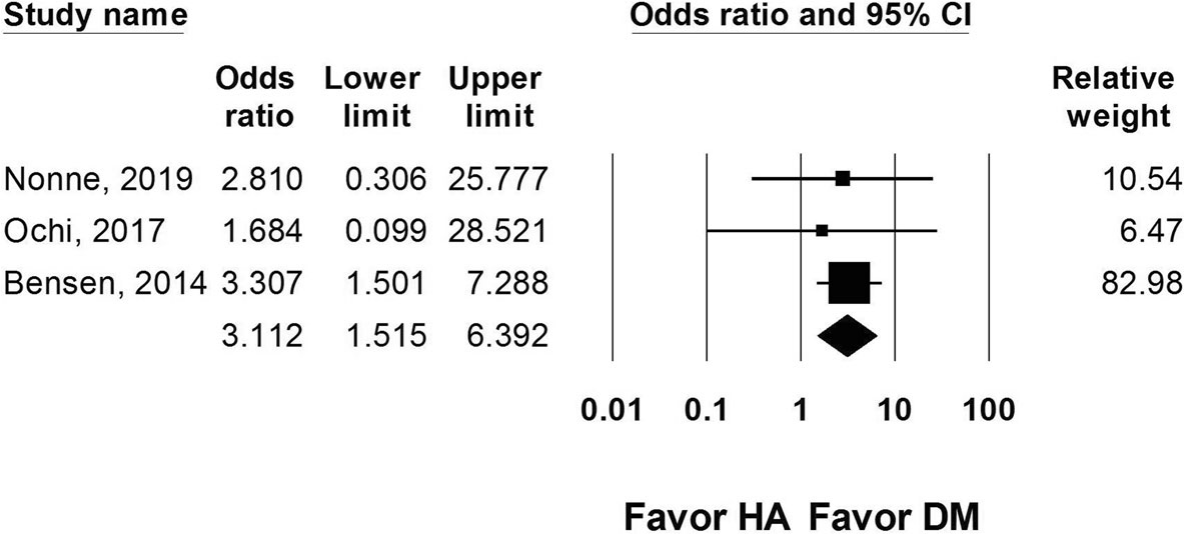
Forest plot comparing implant failure rate after dual-mobility total hip arthroplasty (DM-THA) versus bipolar hemiarthroplasty (HA)

Six studies reported implant failures after DM-THA or HA procedures. Three of the six studies did not have any failures in both groups [10–12]. Therefore, the other three studies were analyzed, with 268 DM-THA and 279 HA procedures included. Data from these two studies showed a higher risk of implant failure in HA group, with an OR of 3.112 (95% CI 1.515 to 6.392, *I*^2^ = 0, Fig. 3).

#### Reoperation rate

**Fig. 4:**
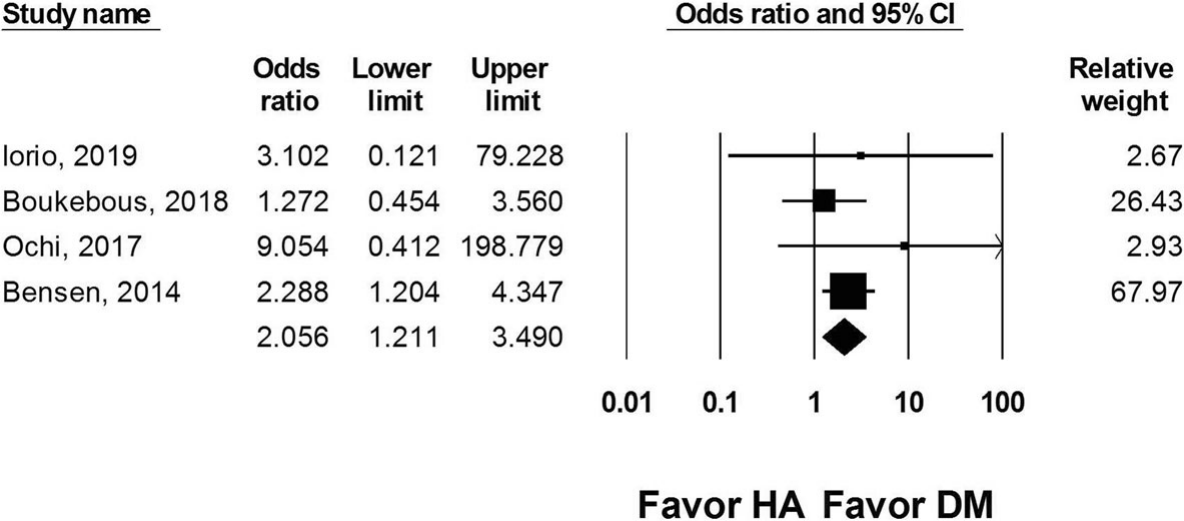
Forest plot comparing reoperation rate after dual-mobility total hip arthroplasty (DM-THA) versus bipolar hemiarthroplasty (HA)

Reoperation rate was reported in four studies. Data were included from 336 DM-THA and 322 HA procedures. The analysis reported a significantly higher reoperation rate after HA than DM-THA (OR: 2.056, 95% CI 1.211 to 3.490, *I*^2^ = 0, Fig. 4). The multivariate regression analysis revealed that age, female sex, posterolateral approach, and choice of DM-THA or HA were not associated with reoperation (Table 3).

#### One-year mortality rate

**Fig. 5:**
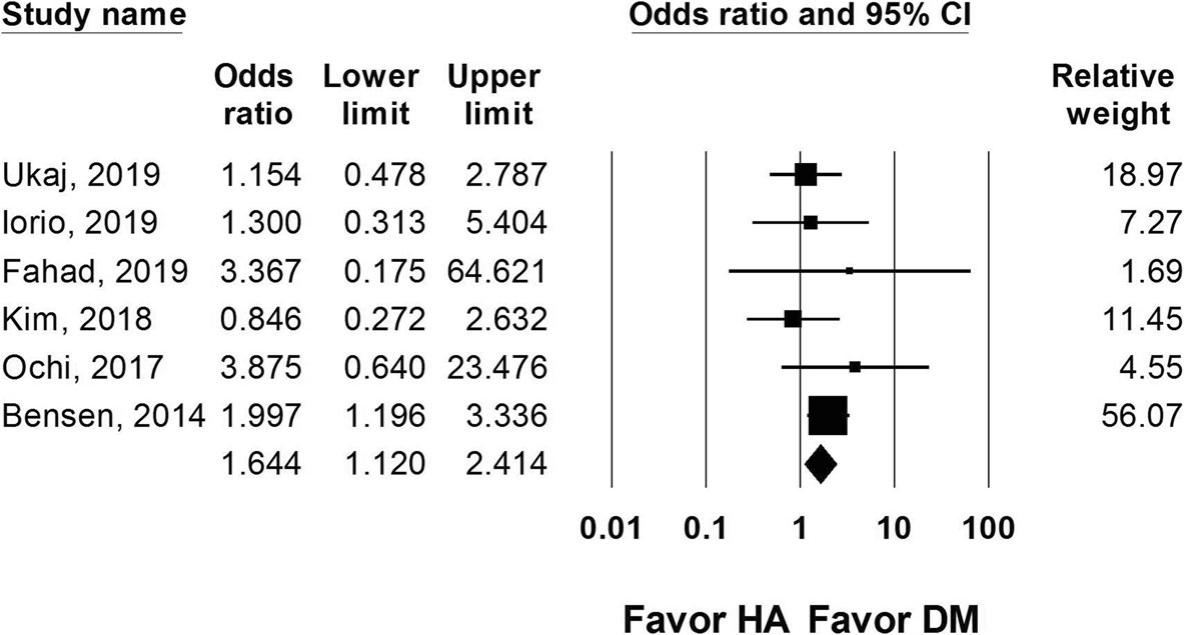
Forest plot comparing 1-year mortality rate after dual-mobility total hip arthroplasty (DM-THA) versus bipolar hemiarthroplasty (HA)

We included all-cause mortality reported within the first year after the index procedure for FNF. Six studies that reported 1-year mortality rate were included, with 396 DM-THA and 431 HA procedures. Data from these six studies showed a higher 1-year mortality rate in the HA group (OR 1.644, 95% CI 1.120 to 2.414, *I*^2^ = 0, Fig. 5).

#### Harris hip score

**Fig. 6:**
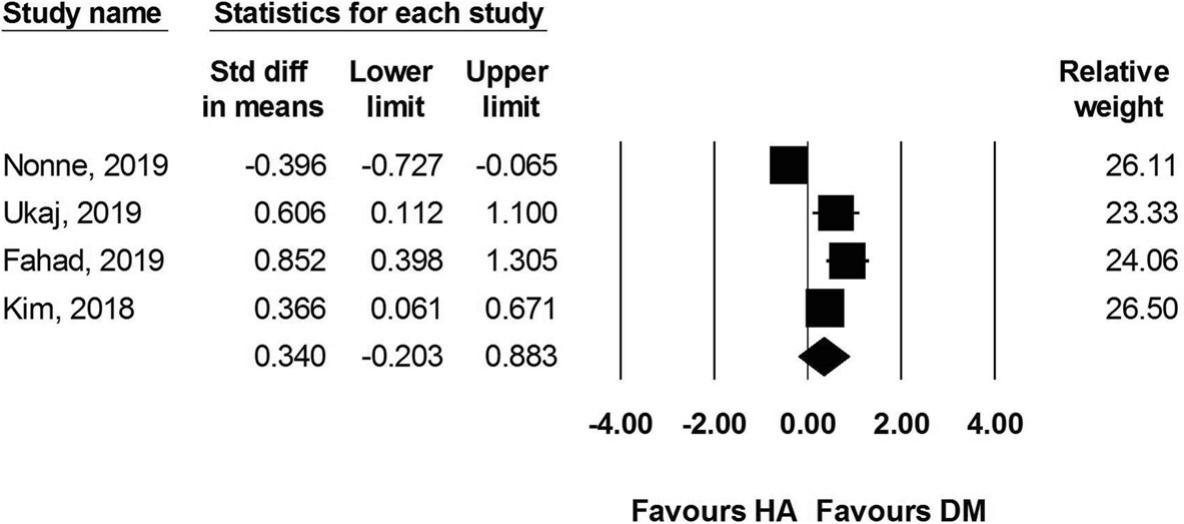
Forest plot comparing Harris hip score after dual-mobility total hip arthroplasty (DM-THA) versus bipolar hemiarthroplasty (HA)

Four studies reported HHS at the postoperative follow-ups. A total of 205 DM-THA and 281 HA procedures were included. The results showed no difference between the two groups (SMD 0.340, 95% CI − 0.203 to 0.883; *I*^2^:87%, Fig. 6).

#### Operation time

**Fig. 7:**
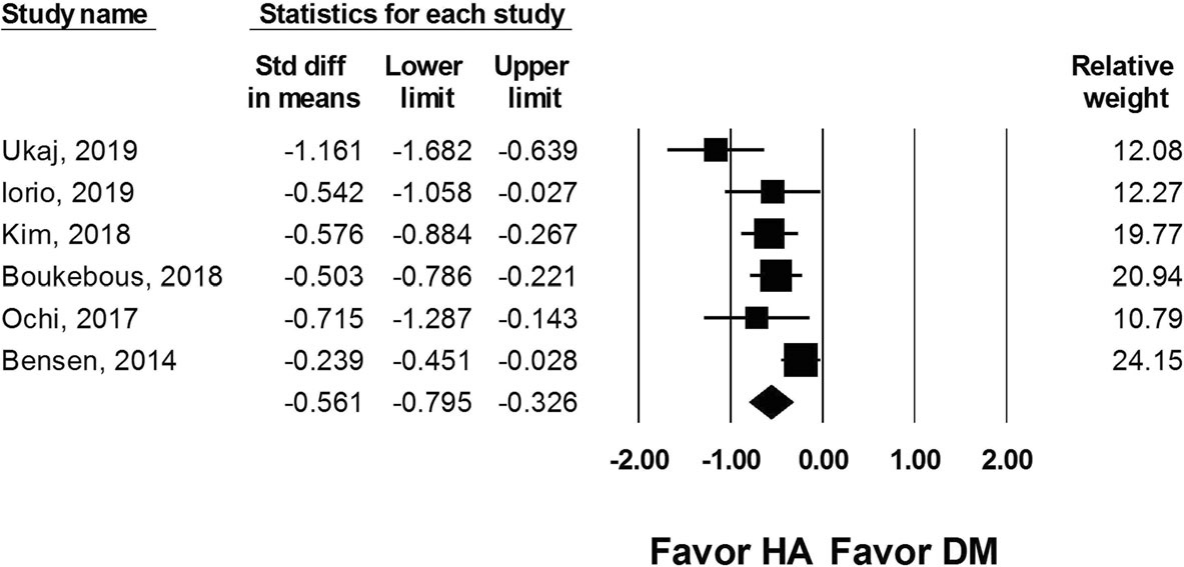
Forest plot comparing operation time after dual-mobility total hip arthroplasty (DM-THA) versus bipolar hemiarthroplasty (HA)

Six studies (454 DM-THA and 438 HA procedures) reported the operation time. The operation time was shorter for patients who received a HA procedure (SMD − 0.561, 95% CI − 0.795 to − 0.326, *I*^2^ = 60.3%, Fig. 7).

#### Intraoperative blood loss

**Fig. 8:**
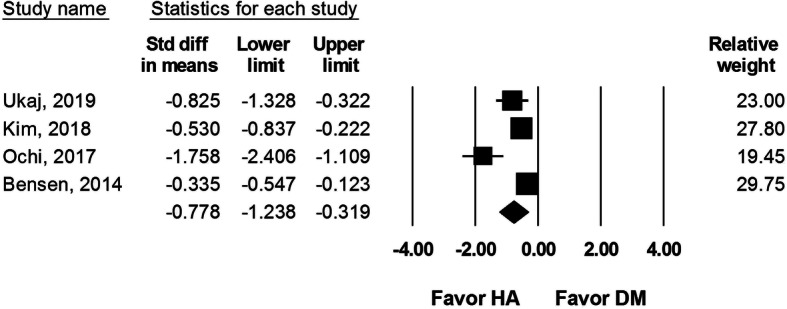
Forest plot comparing intraoperative blood loss after dual-mobility total hip arthroplasty (DM-THA) versus bipolar hemiarthroplasty (HA)

Intraoperative blood loss was reported in three studies, including 326 DM-THA and 307 HA procedures. The analysis reported significantly less intraoperative blood loss after HA than DM-THA procedure (SMD − 0.778, 95% CI − 1.238 to − 0.319, *I*^2^ = 83.6%, Fig. 8).

#### Publication bias

No funnel plot asymmetry which explored the publication bias was detected in terms of the effect sizes of DM-THA versus HA for the aforementioned results (Fig S1, S2, S3, S4, S5, S6 and S7).

## Discussion

In this meta-analysis, we compared the outcome after DM-THA and HA in patients with FNF. We reviewed 2 RCTs and 6 retrospective comparative studies including 541 DM-THA and 603 HA procedures. DM-THA was associated with a lower dislocation rate (OR 3.599; 95% CI 1.954 to 6.630), a lower reoperation rate (OR 2.056; 95% CI 1.211 to 3.490) but an increased operation time (SMD − 0.561; 95% CI − 0.795 to -0.326) and intraoperative blood loss (SMD − 0.778; 95% CI − 1.238 to − 0.319). In multivariate linear regression analysis, age, female sex, posterolateral approach, and choice of DM-THA or HA were not associated with dislocation or reoperation.

HA is one of the main treatment options for patients with a senile FNF. Dislocation after a HA procedure for senile FNF continues to be an important clinical issue with reported incidence from 1 to 15% [18–23]. The various reported incidence might result from different surgical approaches, surgical techniques, or pelvic morphologic features [20, 21]. Despite this heterogeneity, these patients shared some common characteristics including advanced age and a substantial proportion of cognitive disorder or dementia, which increased the risk of postoperative dislocations [8, 20–22]. Most of the dislocations occur within 1 month after surgery. A revision procedure is usually required since close reduction is generally unsuccessful [23].

DM-THA has been utilized in certain patients that are at higher risk for hip dislocations after THA. The dislocation rate in primary THA (0.46%) and revision THA (2.2%) is considered relatively low [4]. Compared with the standard THA, the design of DM-THA and HA increases effective head size and head-to-neck ratio, which increases range of motion and lower the risk of dislocation [24–26]. In this study, the risk of dislocation was lower in DM-THA than HA after FNF (OR 3.599, 95% CI 1.954 to 6.630). Despite the increased effective head size, the stability of HA may also be affected by other factors, including pelvic morphologic features (e.g., acetabular under-coverage or femoral head extrusion) which can also increase the risk of dislocation [20]. The head coverage of DM-THA is based on the size of the cup and shell, which is not affected by native pelvis morphology. Compared with the HA group, the reoperation rate was also lower (OR 2.056; 95% CI 1.211 to 3.490) in the DM-THA group, which can partly be explained by the decreased dislocation rate [11, 14, 15]. However, two included studies that account for large relative weights of the analysis were biased with the indication for DM-THA and HA [14, 15]. In one retrospective study, patients with displaced FNF with osteoarthritis were treated with DM-THA and displaced FNF without osteoarthritis treated with HA from year 2007 to 2008. DM-THA were performed in all patients with displaced FNF since 2009 but not all the surgeons followed this strategy. The age in the HA group were older than the DM-THA group (84.1 vs. 75.2 years). In addition, the inferior results of HA might be biased with a higher proportion of patients being operated by junior surgeons than that of DM-THA [15]. In another retrospective study, surgeons selected DM-THA for patients with FNF and in good general condition or with osteoarthritis and HA in older patients with FNF. Compared with the DM-THA group, patients in the HA group were older, with lower activity levels and higher proportion of dementia. The authors concluded that the benefits of a lower dislocation and reoperation rate from DM-THA only existed in patients with more complicated comorbidities and a dependent status [14]. Two meta-analyses concluded that DM-THA was an option for patients with displaced FNF with lower dislocation and reoperation rate [27, 28]. However, because of biased patient characteristics such as age, we performed multivariate regression analysis, which revealed that age, female sex, surgical approach, and choice of DM-THA or HA were not associated with dislocation or reoperation. The advantages claimed for DM-THA over HA were limited. The advantages might be significant in high-risk population, such as cognitive disorder or dementia [20–22], which was included in only two of the eight studies being analyzed [9, 11].

Acetabular erosion and stem loosening have been reported to be two most common causes of failure after a HA procedure. The mean time from index surgery to those symptomatic failures ranged from 34 to 96 months [29]. A modern generation of DM-THA has been reported with excellent long-term implant survival (more than 95% at 10-year follow-up) [30]. Since the main objective for most of the included studies was to compare short-term outcome domains, including dislocation, reoperation, functional score, and mortality, the mean follow-up duration ranged from only 12 to 36 months, which was too short to validate and compare implant survival between DM-THA and HA group. With regard to intraoperative parameters, DM-THA was associated with a longer operation time and more intraoperative blood loss than HA. This difference is expected because of the additional acetabular preparation in the DM-THA procedure. However, the difference in operation time (DM-THA vs. HA, 77.5 vs. 66.7 min) and intraoperative blood loss (DM-THA vs. HA, 458.2 vs. 333.0 ml) did not show an effect on some important outcome domains such as need of transfusion or mortality [10, 11, 13]. Our pooled results showed an increased 1-year mortality rate in the HA group. However, the only study that found a difference in 1-year mortality between DM-THA and HA group (DM-THA vs. HA, 17.1% vs. 29.2%) might be biased since the HA group consisted of patients that were relatively older (DM-THA vs. HA, 75.2 vs. 84.1 years) [15].

There are some limitations should be emphasized. First, we only included studies written in English but not in other languages or unpublished data. This might lead to a potential publication bias. Second, we included both RCTs and retrospective comparative studies. Some studies have pointed out potential biases including age, activity, and mobility level, dependent status or surgeons’ choice of procedure based on their experience and familiarity which all can have potential biases due to the nature of the study design [9, 14, 15]. Third, there is heterogeneity in the patient characteristics between the studies (e.g., age, sex, comorbidity index, activity level, surgeons’ experience, surgical approaches, and types of implants).

## Conclusions

Based on the current evidence, the advantages reported for DM-THA over HA with regard to dislocation and reoperation rate in elderly patients with FNF remain inconclusive because many of the included studies were limited by biased selection criteria or included all patients with FNF rather than high-risk population such as patients with cognitive disorder or dementia. High-quality studies on these high-risk patients are warranted to validate the value of DM-THA.

## Supplementary Information

**Additional file 1: Figure S1.** Funnel plot of dislocation rate.

**Additional file 2: Figure S2.** Funnel plot of implant failure rate.

**Additional file 3: Figure S3.** Funnel plot of reoperation rate.

**Additional file 4: Figure S4.** Funnel plot of one-year mortality rate.

**Additional file 5: Figure S5.** Funnel plot of Harris hip score.

**Additional file 6: Figure S6.** Funnel plot of operation time.

**Additional file 7: Figure S7.** Funnel plot of intraoperative blood loss.

## Data Availability

The information to access the data in the study is included within this article.

## Abbreviations

DM: Dual mobility
FNF: Femoral neck fracture
HA: Hemiarthroplasty
HHS: Harris hip score
OR: Odds ratio
RCT: Randomized controlled trial
SMD: Standardized mean difference
THA: Total hip arthroplasty
