# Supplementary figures and images for: Outcomes of dual-mobility total hip arthroplasty versus bipolar hemiarthroplasty for patients with femoral neck fractures: a systematic review and meta-analysis
Source: J Orthop Surg Res. 2021 Feb 24;16:152. doi: 10.1186/s13018-021-02316-6 (PMC7903652; doi:10.1186/s13018-021-02316-6)

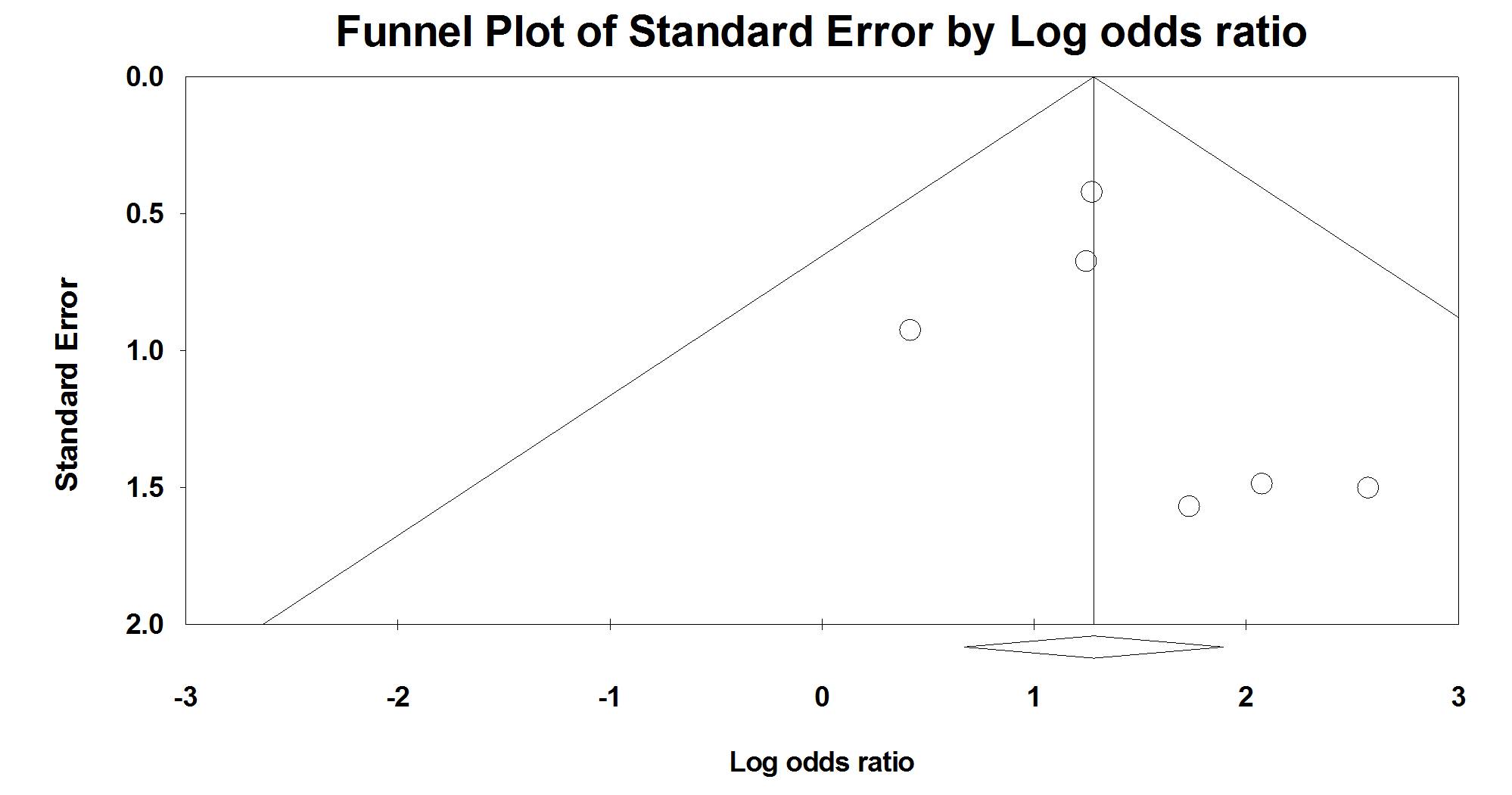

Supplement: Supplementary file 1 — Additional file 1: Figure S1. Funnel plot of dislocation rate. [file 13018_2021_2316_MOESM1_ESM.jpg]

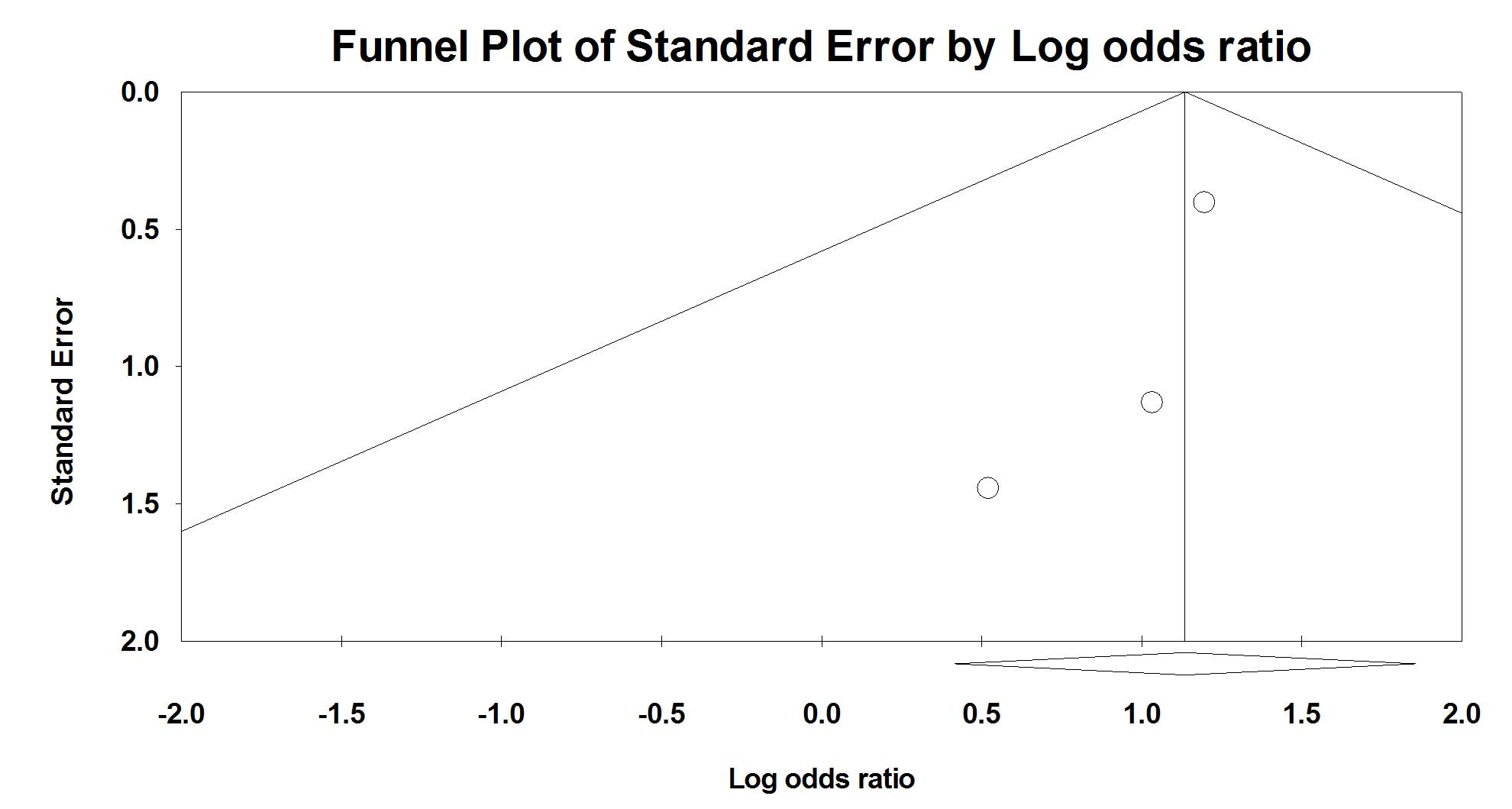

Supplement: Supplementary file 2 — Additional file 2: Figure S2. Funnel plot of implant failure rate. [file 13018_2021_2316_MOESM2_ESM.jpg]

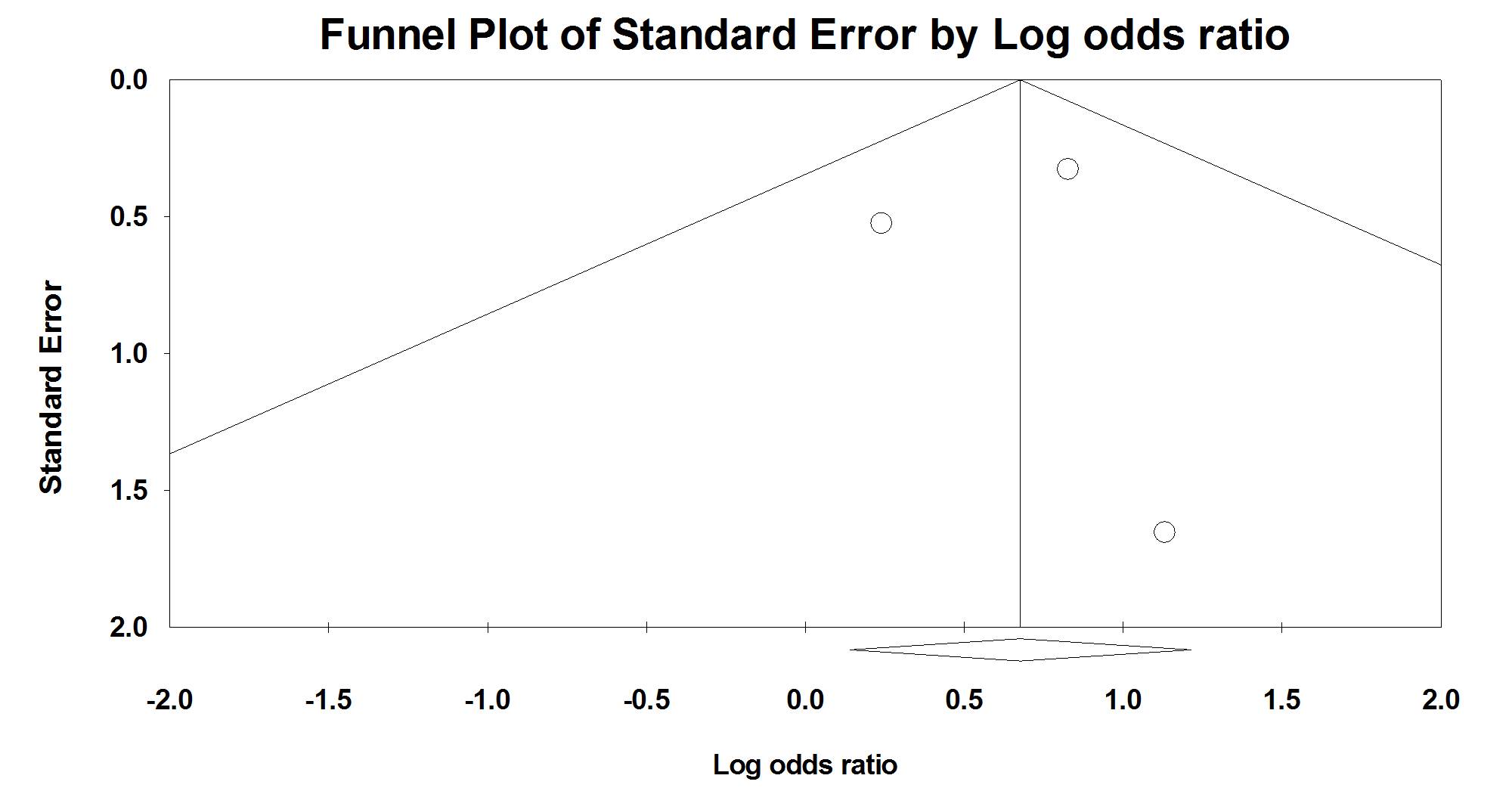

Supplement: Supplementary file 3 — Additional file 3: Figure S3. Funnel plot of reoperation rate. [file 13018_2021_2316_MOESM3_ESM.jpg]

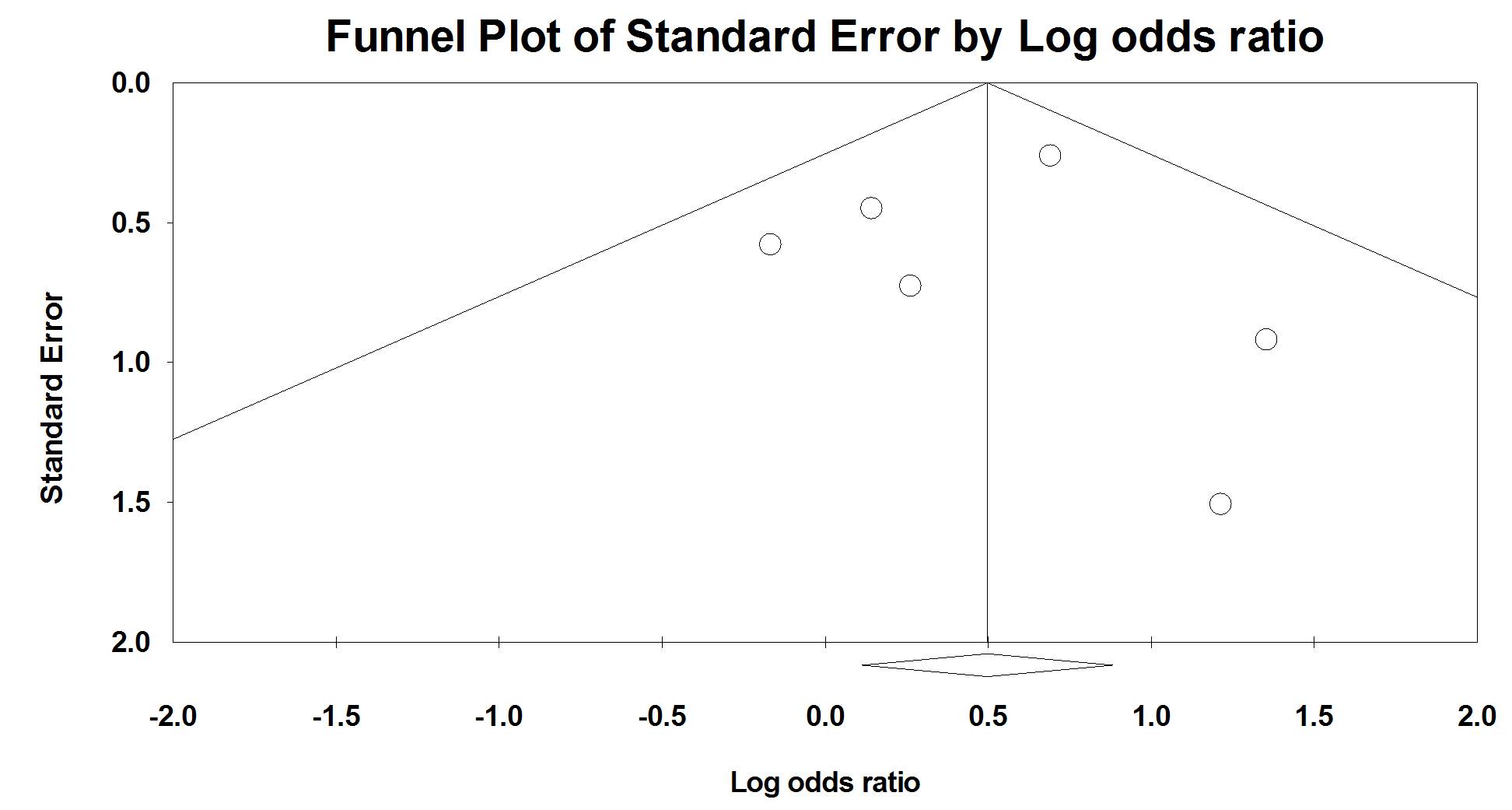

Supplement: Supplementary file 4 — Additional file 4: Figure S4. Funnel plot of one-year mortality rate. [file 13018_2021_2316_MOESM4_ESM.jpg]

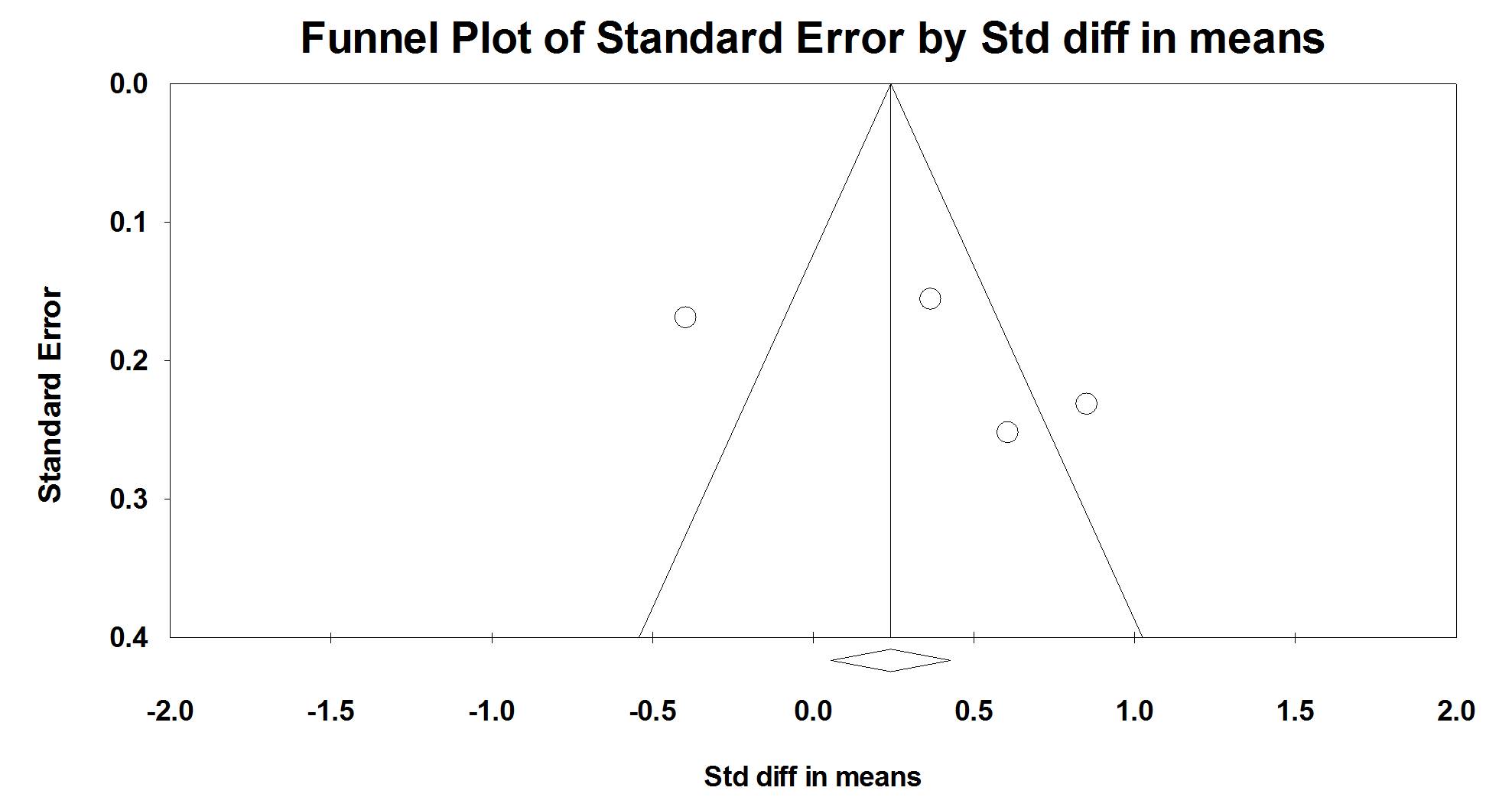

Supplement: Supplementary file 5 — Additional file 5: Figure S5. Funnel plot of Harris hip score. [file 13018_2021_2316_MOESM5_ESM.jpg]

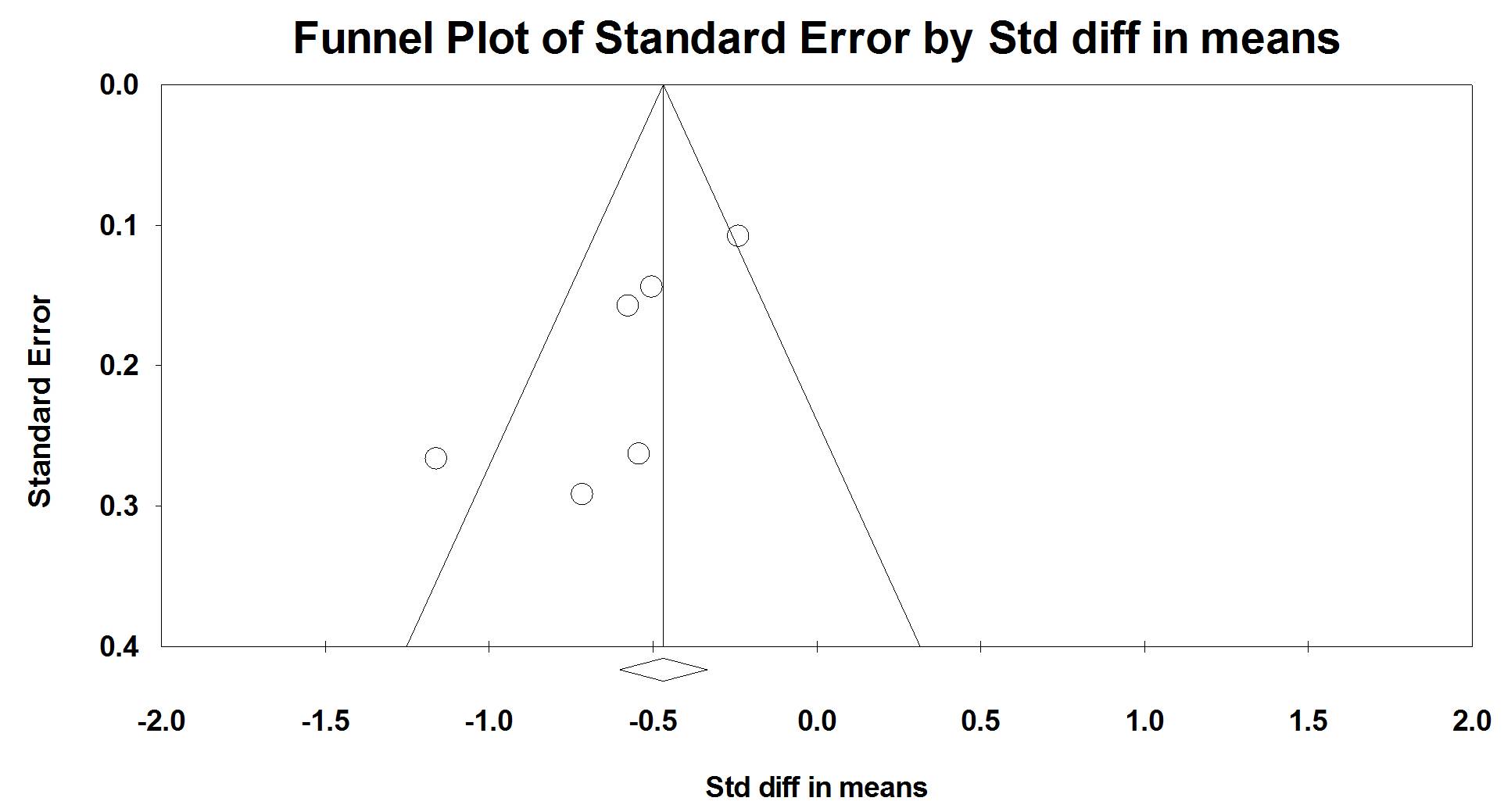

Supplement: Supplementary file 6 — Additional file 6: Figure S6. Funnel plot of operation time. [file 13018_2021_2316_MOESM6_ESM.jpg]

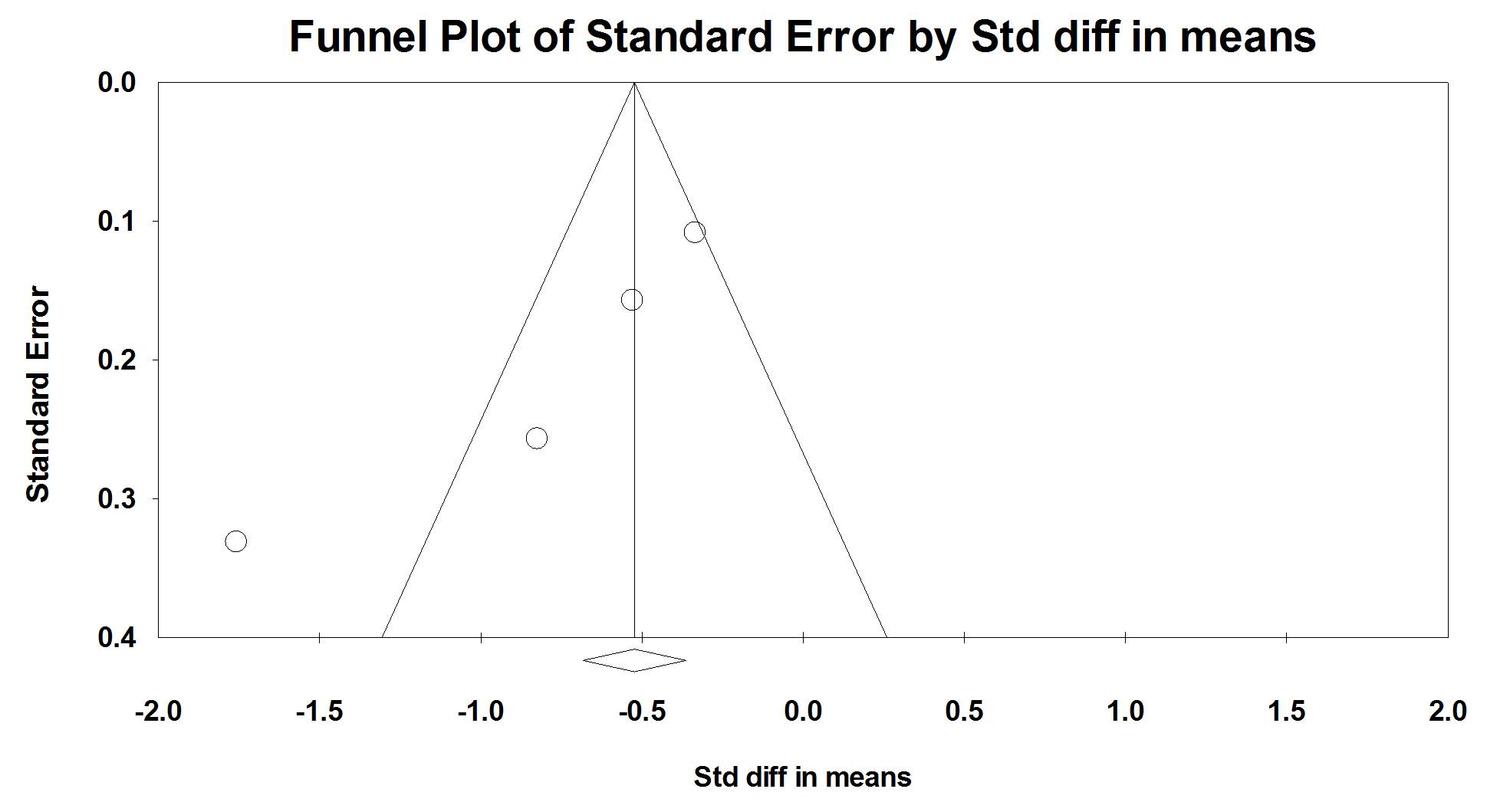

Supplement: Supplementary file 7 — Additional file 7: Figure S7. Funnel plot of intraoperative blood loss. [file 13018_2021_2316_MOESM7_ESM.jpg]
